# A PHENOMENOLOGICAL PERSPECTIVE OF INTERGENERATIONAL ENGAGEMENT, SOCIAL ISOLATION, AND AGE SEGREGATION

**DOI:** 10.1093/geroni/igz038.550

**Published:** 2019-11-08

**Authors:** Andrew Steward, Kimberly McDevitt

**Affiliations:** University of Denver, Denver, Colorado, United States

## Abstract

Intergenerational engagement and its impact on social isolation is an area of critical importance within the broader topic of healthy aging. This phenomenological study involved individual qualitative interviews with older adults (60+ years of age without cognitive impairment). Participants (N=9) were recruited using fliers and referrals from assisted living staff. Purposive sampling was utilized, and researchers attempted to diversify the sample by selecting participants from multiple settings and cultural backgrounds. This study was guided by two overarching research aims: 1) To understand the nature of intergenerational engagement and its impact on reducing social isolation, and 2) To understand how intergenerational experiences have changed over time. Interviews were conducted by the study PI, lasted 60-minutes and took place in participants’ homes or agreed-upon public settings. Two researchers (the PI and an MSW intern) participated in the data collection and analysis process, which helped strengthen inter-coder reliability. Two important themes emerged from the data. First, participants often described intergenerational engagement as stimulating and stated that they enjoy feeling “younger” or “childlike” when engaging with younger generations. Second, participants reflected that intergenerational connections were more naturally built into their communities as young people and that society today appears to be increasingly age-segregated. Further development of internalized ageism measures may lead to a better understanding of both the positive and negative aspects of internalized ageism. Future studies should also explore, from the perspective of older adults, recommendations for re-building intentionally intergenerational communities that facilitate authentic engagement and enhanced well-being for all generations.

